# Identification of Ebola Virus Inhibitors Targeting GP2 Using Principles of Molecular Mimicry

**DOI:** 10.1128/JVI.00676-19

**Published:** 2019-07-17

**Authors:** Courtney D. Singleton, Monica S. Humby, Hyun Ah Yi, Robert C. Rizzo, Amy Jacobs

**Affiliations:** a Department of Molecular & Cellular Pharmacology, Stony Brook University, Stony Brook, New York, USA; b Department of Microbiology and Immunology, School of Medicine and Biomedical Sciences, State University of New York (SUNY) at Buffalo, Buffalo, New York, USA; c Department of Applied Mathematics & Statistics, Stony Brook University, Stony Brook, New York, USA; d Institute of Chemical Biology & Drug Discovery, Stony Brook University, Stony Brook, New York, USA; e Laufer Center for Physical & Quantitative Biology, Stony Brook University, Stony Brook, New York, USA; University of Kentucky College of Medicine

**Keywords:** DOCK, EBOV, computer-aided drug design, docking, footprint similarity, membrane fusion, molecular dynamics, viral entry, virtual screening

## Abstract

A key step in the Ebola virus (EBOV) replication cycle involves conformational changes in viral glycoprotein 2 (GP2) which facilitate host-viral membrane fusion and subsequent release of the viral genome. Ebola GP2 plays a critical role in virus entry and has similarities in mechanism and structure to the HIV gp41 protein for which inhibitors have been successfully developed. In this work, a putative binding pocket for the C-terminal heptad repeat in the N-terminal heptad repeat trimer was targeted for identification of small molecules that arrest EBOV-host membrane fusion. Two computational structure-based virtual screens of ∼1.7 M compounds were performed (DOCK program) against a GP2 five-helix bundle, resulting in 165 commercially available compounds purchased for experimental testing. Based on assessment of inhibitory activity, cytotoxicity, and target specificity, four promising candidates emerged with 50% inhibitory concentration values in the 3 to 26 μM range. Molecular dynamics simulations of the two most potent candidates in their DOCK-predicted binding poses indicate that the majority of favorable interactions involve seven highly conserved residues that can be used to guide further inhibitor development and refinement targeting EBOV.

**IMPORTANCE** The most recent Ebola virus disease outbreak, from 2014 to 2016, resulted in approximately 28,000 individuals becoming infected, which led to over 12,000 causalities worldwide. The particularly high pathogenicity of the virus makes paramount the identification and development of promising lead compounds to serve as inhibitors of Ebola infection. To limit viral load, the virus-host membrane fusion event can be targeted through the inhibition of the class I fusion glycoprotein of *Ebolavirus*. In the current work, several promising small-molecule inhibitors that target the glycoprotein GP2 were identified through systematic application of structure-based computational and experimental drug design procedures.

## Abstract (precis)

The most recent Ebola virus disease outbreak, from 2014 to 2016, resulted in approximately 28,000 individuals becoming infected, which led to over 12,000 causalities worldwide. The particularly high pathogenicity of the virus makes paramount the identification and development of promising lead compounds to serve as inhibitors of Ebola infection. To limit viral load, the virus-host membrane fusion event can be targeted through the inhibition of the class I fusion glycoprotein of *Ebolavirus*. In the current work, several promising small-molecule inhibitors that target the glycoprotein GP2 were identified through systematic application of structure-based computational and experimental drug design procedures.

## INTRODUCTION

Ebola virus disease (EVD), a severe hemorrhagic fever caused by specific Ebola viruses, was first documented in West Africa in 1976 (1). During the most recent outbreak (2014 to 2016), EVD spread across West Africa (2), Europe, and the United States, (3), resulting in approximately 12,000 deaths worldwide (4). The most common and also the most pathogenic species, Zaire ebolavirus (EBOV), causes infection with a mortality rate of approximately 90% (5–7). Infected individuals typically present with intense fever, weakness, and gastrointestinal symptoms, including excessive diarrhea (3), abdominal pain, and vomiting (8, 9). The particularly high pathogenicity of the virus (5–7), likelihood of additional naturally occurring outbreaks (10), concern for use as a bioterrorist agent (11), and lack of FDA-approved therapeutics to treat EVD (12) makes the identification of effective therapeutic interventions paramount.

**FIG 1:**
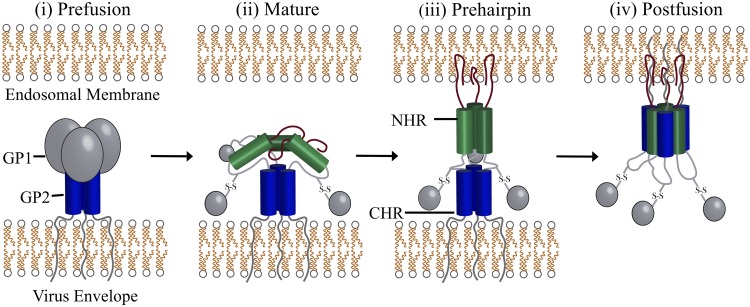
EBOV entry requires a conformational change in GP2. (i) In the prefusion native state, GP1, along with other accessory proteins, surrounds the triple-helix GP2. (ii) Upon GP maturation, the cleaved form of GP2 binds to NPC1. (iii) GP2 extends its internal fusion loop region into the host membrane, forming an extended prehairpin intermediate that is the structure targeted in this work to identify molecules that bind the NHR. (iv) The GP2 CHR helices fold over the NHR trimer, forming a postfusion 6HB, which drives fusion of the host-virus membranes, allowing EBOV to enter the cell (109, 110).

EBOV contains a nonsegmented, single-stranded, negative-sense RNA genome that encodes eight viral proteins: nucleoprotein (NP), polymerase cofactor (VP35), matrix protein (VP40), transcription activator (VP30), matrix protein (VP24), RNA-dependent RNA polymerase (L), and glycoprotein (GP), which is comprised of two proteins, the receptor attachment glycoprotein 1 (GP1) and membrane fusion glycoprotein 2 (GP2) (13, 14). Upon infection, GP1 is involved in host cell recognition/attachment (15) and virus uptake into host cells, primarily through macropinocytosis (16). Inside the cell, the virus is trafficked into the endosome (7, 15), where acidification of the late endosome triggers host cysteine proteases cathepsin L and B (17, 18) to cleave the prefusion form of GP (Fig. 1i) into its mature 19-kDa form (19, 20). For viral entry to occur, GP1 must interact with the Neimann-Pick disease type C1 (NPC1) protein (21–23), the only known fusion receptor for EBOV, which releases GP2 from its conformational constraints (Fig. 1ii). Subsequently, the internal fusion loop of GP2 extends into the endosomal membrane (Fig. 1iii) and GP2 undergoes a conformational change whereby the C-terminal heptad repeat (CHR) folds around the N-terminal heptad repeat (NHR) trimer, forming a six-helix bundle (6HB) (Fig. 1iv) (24). Formation of the 6HB brings the virus and host membrane into close proximity, facilitating membrane fusion, which permits the escape of the EBOV genome from the endosome into the host cell (25).

Previously reported EBOV inhibitors include antibodies, peptides, and small molecules which target different viral and host cell proteins involved in the EBOV replication cycle, including GP (6, 26–37), VP40 (38), NPC1 (22, 30), cathepsin (30, 39), and Hsp90 (40). Focusing on small molecules, prior work has led to the identification of compounds that are believed to (6, 36), or have been shown to (32, 35), interact with a prefusion form of GP which likely destabilizes the complex inhibiting viral entry. To date, however, no researchers have reported small molecules that target the important EBOV prehairpin intermediate (Fig. 1iii), which could prevent formation of the 6HB in a manner exploited by the FDA-approved HIV gp41 inhibitor enfuvirtide (Fuzeon) (41). Several GP2 peptidomimetics have, however, been identified, which provides an important proof of concept (26–28). Due to its key role in EBOV entry and the demonstrated utility of targeting HIV gp41 (an analogous class I fusion protein), GP2 is a promising target for small-molecule rational drug design and the subject of this investigation.

The goal of this study was identification of drug-like small molecules that target a pocket in the NHR of the EBOV GP2 prehairpin intermediate using atomic-level molecular modeling tools and experimental characterization. Specifically, large-scale virtual screening of ∼1.7 million small molecules was performed with GP2 using the program DOCK6 (42). We hypothesize that small molecules that interact with the GP2 NHR pocket will interfere with assembly of the 6HB required for EBOV-host membrane fusion (Fig. 1). Interfering with 6HB formation is a strategy previously employed successfully against HIV (43–52) through targeting an analogous pocket on the viral protein gp41 (53, 54). The computational screening resulted in the prioritization and purchase of 165 compounds for experimental characterization, which led to 11 hits that inhibit viral entry in both EBOV-GP-pseudotyped virus and EBOV transcription- and replication-competent virus-like particle (trVLP) systems. Compounds were further evaluated to assess (i) potential activity artifacts using detergent-containing experiments, (ii) specificity for EBOV using a vesicular stomatitis virus glycoprotein (VSV-G)-pseudotyped virus particle counterscreen, and (iii) step(s) within the EBOV replication cycle where they exerted the majority of inhibitory activity using time-of-addition (TOA) analysis. Results suggest that 4 of the 11 compounds act to specifically inhibit EBOV entry after attachment but prior to virus-host membrane fusion. Molecular dynamics (MD) simulations in conjunction with genome analysis identified 7 highly conserved residues across different Ebola virus strains (E564.A, A568.A, L571.A, F572.A, T566.C, L569.C, and L573.C) that contribute a majority of the favorable interactions between the compounds and GP2.

## RESULTS

### Virtual screening outcomes.

**FIG 2:**
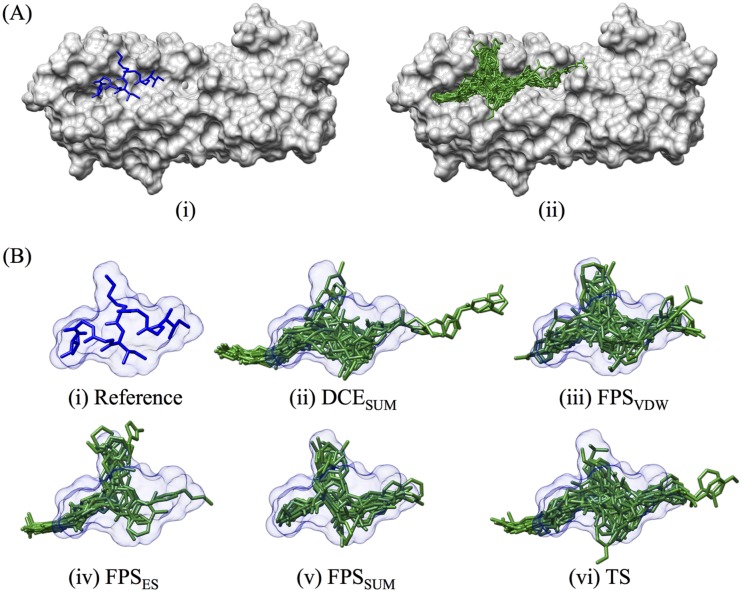
Visualization of the purchased docked molecules. (A) The reference peptide (in blue, i) and all 83 purchased molecules (in green, ii) and how they fit in the surface of the EBOV five-helix bundle, in gray. (Bi) Reference ligand and surface (in blue). Overlap of the 83 purchased molecules was based on all five ranked methods, in the following order: (ii) DCE_SUM_ (*n* = 24), (ii) FPS_VDW_ (*n* = 17), (iii) FPS_ES_ (*n* = 12), (iv) FPS_SUM_ (*n* = 10), and (v) TS (*n* = 20). The reference peptide and its surface are in blue. The overlaid purchased compounds are in green.

The goal of this study was to identify molecules that inhibit EBOV infection by interfering with the interactions required for formation of the GP2 six-helix bundle (6HB). Since the conformational change required to produce the postfusion structure is dependent on CHR binding the NHR region of GP2 (Fig. 1), a virtual screen of approximately 1.7 million compounds was conducted to a five-helix bundle model of GP2 constructed by the removal of one CHR from a high-resolution postfusion structure (PDB entry 2EBO [25]) (see Materials and Methods, below). Compound prioritization led to 83 candidates purchased for experimental testing (Fig. 2) and employed five distinct scoring functions: DCE_SUM_ (DOCK Cartesian van der Waals and electrostatic energy), FPS_VDW_ (footprint comparison of the van der Waals energy of the reference peptide and selected ligands), FPS_ES_ (footprint for electrostatic energy), FPS_SUM_ (footprint for both van der Waals and electrostatic energy), and TS (total score; the combination of DCE_SUM_ and FPS_SUM_). A large number of molecules was prioritized based on their structural and spatial similarity to the reference ligand composed of a segment of the CHR that made the most favorable interactions with our model of a GP2 five-helix bundle. As a rule, all 83 compounds chosen for experimental testing showed good overlap with the reference (Fig. 2A). However, those selected based on favorable footprint similarity (FPS) have somewhat better overlap than those selected based on DCE or TS (Fig. 2B).

Consistent with visual inspection (Fig. 2), molecules in each of the five groups share similar size and flexibility, with a mean molecular weight (MW) distribution of 467.3 g/mol and number of rotatable bonds of 9.5 (Table 1). Compounds purchased based on similarity in electrostatic (ES) interaction profiles (FPS_ES_) had the overall smallest MW (414.0 g/mol) and fewer numbers of rotatable bonds (8.3), while those selected from the TS list were largest (492.3 g/mol) (Table 1). As expected (45, 55), compounds selected using a specific scoring function (Table 1, scoring function column) generally showed the best average score with regard to that specific chemical or physical property (Table 1, Property columns). For example, compounds prioritized using the DCE_SUM_ function yielded a more favorable (lower) average DCE_SUM_ energy (−65 kcal/mol) than those obtained using other functions (−49 to −59 kcal/mol). Likewise, molecules selected using FPS_SUM_ resulted in a more favorable average FPS_SUM_ score (5.5) than the other groups (7.8 to 19.1). For compounds prioritized using FPS_ES_ and FPS_VDW_ footprint components, the scores were the lowest (1.6) and second lowest (3.9), respectively, among their respective FPS_ES_ and FPS_VDW_ groups.

**TABLE 1:** Summary of ligand properties from the initial computational screen

Scoring function^*a*^	Property^*b*^								
	*N*	MW	RB	DCE_SUM_	DCE_VDW_	DCE_ES_	FPS_SUM_	FPS_VDW_	FPS_ES_
DCE_SUM_	24	460.9 ± 41.8	9.2 ± 2.0	−65.0 ± 5.9	−49.5 ± 3.6	−15.5 ± 5.4	19.1 ± 2.2	8.8 ± 1.5	10.3 ± 2.3
FPS_SUM_	10	479.6 ± 30.2	9.8 ± 1.8	−49.3 ± 0.9	−46.5 ± 1.2	−2.7 ± 0.9	5.5 ± 0.6	3.1 ± 0.4	2.4 ± 0.5
FPS_VDW_	17	477.2 ± 34.4	9.4 ± 1.5	−49.8 ± 1.7	−46.1 ± 2.0	−3.6 ± 2.0	7.8 ± 2.3	3.9 ± 0.9	3.9 ± 1.8
FPS_ES_	12	414.0 ± 45.6	8.3 ± 1.4	−48.7 ± 0.9	−45.3 ± 1.4	−3.3 ± 1.0	8.6 ± 1.5	6.9 ± 1.6	1.6 ± 0.2
TS	20	492.3 ± 19.4	10.7 ± 1.6	−59.1 ± 3.1	−55.6 ± 2.2	−3.5 ± 3.1	10.5 ± 2.5	6.8 ± 1.0	3.7 ± 1.9
Cum avg	83	467.3 ± 42.5	9.5 ± 1.8	−56.2 ± 7.8	−49.3 ± 4.6	−6.9 ± 6.5	11.5 ± 5.4	6.3 ± 2.4	5.0 ± 3.8

a Abbreviations: DCE_SUM_ (DOCK Cartesian van der Waals and electrostatic energy), FPS_VDW_ (footprint comparison of the van der Waals energy of the reference peptide and selected ligands), FPS_ES_ (footprint for electrostatic energy), FPS_SUM_ (footprint for both van der Waals and electrostatic energy), TS (total score, the combination of DCE_SUM_ and FPS_SUM_), cum avg (cumulative average and standard deviation of the total number of compounds or the number of compounds, as appropriate).

b *N* represents the number of molecules in each category. MW, molecular weight (g/mol); RB, rotatable bonds. The values in each column correspond to the means and standard deviations for each descriptor. Energy scores (DCE_SUM_ and DCE_ES_) are computed in kcal/mol, and FPS scores are calculated using the Euclidian distance between the energies of the ligands and reference.

For the DCE_SUM_-selected group, the favorable scores can be attributed to strong ES interactions resulting in an average DCE_ES_ score of −15.5 kcal/mol, over 2-fold greater than the ensemble average (−6.9 kcal/mol). The overall strength of the DCE_SUM_ scores, in conjunction with being the second smallest group in terms of MW and number of rotatable bonds (9.2), suggests that the DCE_SUM_ list compounds are highly polar. In contrast, the TS list interactions are dominated by strong VDW interactions due to their larger size (MW = 492 g/mol) (Table 1). Consistent with the fact that FPS_SUM_ is a part of the TS scoring function, the FPS score components are better than those observed using DCE_SUM_. However, the overlap is relatively moderate (FPS_SUM_ = 10.5, FPS_VDW_ = 6.8, FPS_ES_ = 3.7); therefore, future work could explore increasing the contribution of the FPS component of TS. In summary, molecular property analysis confirms that the 83 purchased candidates are similar in size and flexibility but diverse in terms of interaction energy and overlap the reference peptide.

### Nine molecules from the initial *in silico* screen inhibit EBOV-pseudotyped virus entry *in vitro*.

**FIG 3:**
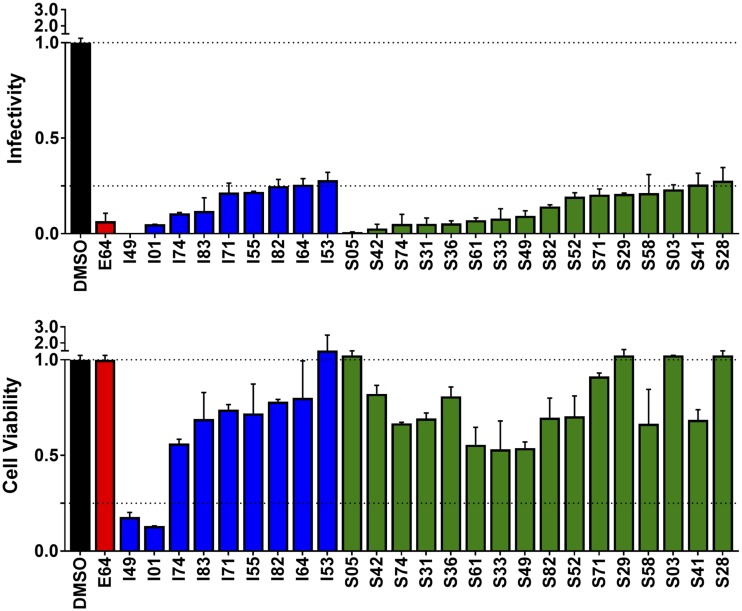
Normalized infectivity and cell viability for the top 25 out of 165 compounds tested against EBOV-GP-pseudotyped virus. Luminescence (top) associated with EBOV-GP-pseudotyped particle entry into 293T cells was measured and normalized by the DMSO control, and cytotoxicity (bottom) was obtained by fluorescence. The negative control (DMSO) is shown in black, and the positive control for inhibition (E64), tested at 50 μM, is shown in red. Molecules from the initial screen (I), in blue, and similarity screen (S), in green, with normalized luciferase signal of ≤0.25, are shown. For both screens, candidate compounds were tested in duplicate at 25 μM.

The 83 compounds identified from the aforementioned *in silico* screen were tested for their ability to inhibit EBOV entry and for cytotoxicity at 25 μM (6, 30, 45). EBOV (HIV-1/EBOV)-pseudotyped virus entry into 293T cells was quantified by luciferase signal normalized by cytotoxicity and dimethyl sulfoxide (DMSO) control to yield the infectivity signal per cell as a fraction of the maximum (see Materials and Methods). Encouragingly, nine compounds resulted in a normalized luciferase signal of ≤0.25 (Fig. 3, blue). Additionally, the observed luciferase signal for the nine compounds was approximately 1.5 standard deviations below the average infectivity signal for all 83 purchased molecules, 0.76 ± 0.40. Although the two compounds with the most activity (I01 and I49) were also the most cytotoxic (Fig. 3, lower, blue), all nine hits with activity were retained and used as starting points for identification of structurally related analogs in a secondary computational screen (see Discussion).

### Secondary similarity screen.

To identify additional compounds with enhanced activity, a second similarity-based computational screen was conducted to explore the chemical search space around the nine initial hits. Each of the hits in turn was used to rescore and rerank the top 100,000 docked molecules from the initial screen to identify compounds with similar functionality and three-dimensional (3D) shape using the DOCK Hungarian similarity (HMS) scoring function (56). The 500 top-scoring molecules from the nine unique lists were further interrogated using five additional functional methods to assess energy score (DCE) and similarity to the initial hit (footprint [FPS], pharmacophore [FMS], volume overlap [VOS], and Tanimoto).

**FIG 4:**
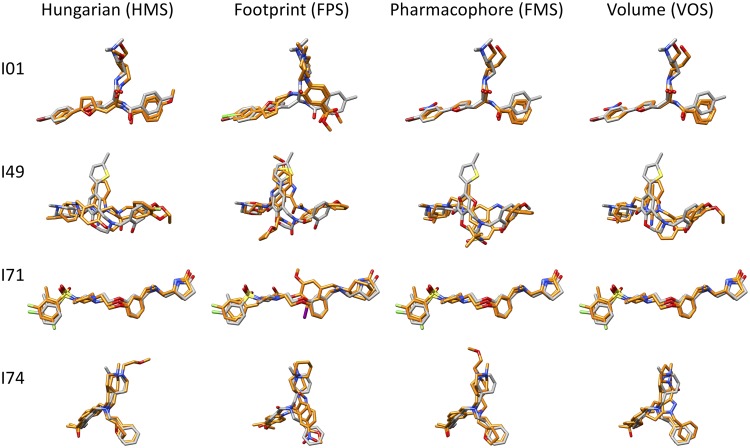
Comparison of docked poses between four compounds from the initial screen (I) with representative candidates selected from the similarity screen (S). Two top-scoring compounds selected from rescoring the virtual screening library with Hungarian (HMS), footprint (FPS), pharmacophore (FMS), or volume overlap similarity (VOS) are shown in orange overlaid with I01, I49, I71, and I74 (gray) from the initial screen in their predicted binding pose.

Figure 4 compares docked geometries for four of the initial hits (gray) overlaid with two representative compounds each (orange) from the secondary screen. In these examples, with the exception of I49, the compounds generally showed strong overlap and made residue-based interaction patterns similar to those of their respective references (Fig. 4), corresponding to a high average VOS score of ∼0.7 and a low average FPS score of ∼5.6. Despite the overall similarity of ligand scaffolds within each group, the use of different DOCK functions generally resulted in the selection of chemically diverse molecules at the atomic level. In some cases, however, the same ligand was the top-ranked candidate across the different groups. For example, rank ordering by pharmacophore or volume overlap yielded the same top-scored results for I01 (FMS = 1.56, VOS = 0.82), which suggests high structure and functional similarity with the initial hit (Fig. 4, FMS and VOS). Overall, the secondary virtual screen resulted in the selection of 82 additional candidates, which were subsequently evaluated for inhibition and cytotoxicity at 25 μM against EBOV-pseudotyped virus. A luciferase signal of ≤0.25, which was more than 1 standard deviation below the population mean luciferase signal of 0.54 ± 0.30, was used to identify 16 additional hits with moderate to low cytotoxicity (Fig. 3, green, S prefix).

### Dose-response characterization of candidates against HIV/EBOV-GP-pseudotyped virus.

**FIG 5:**
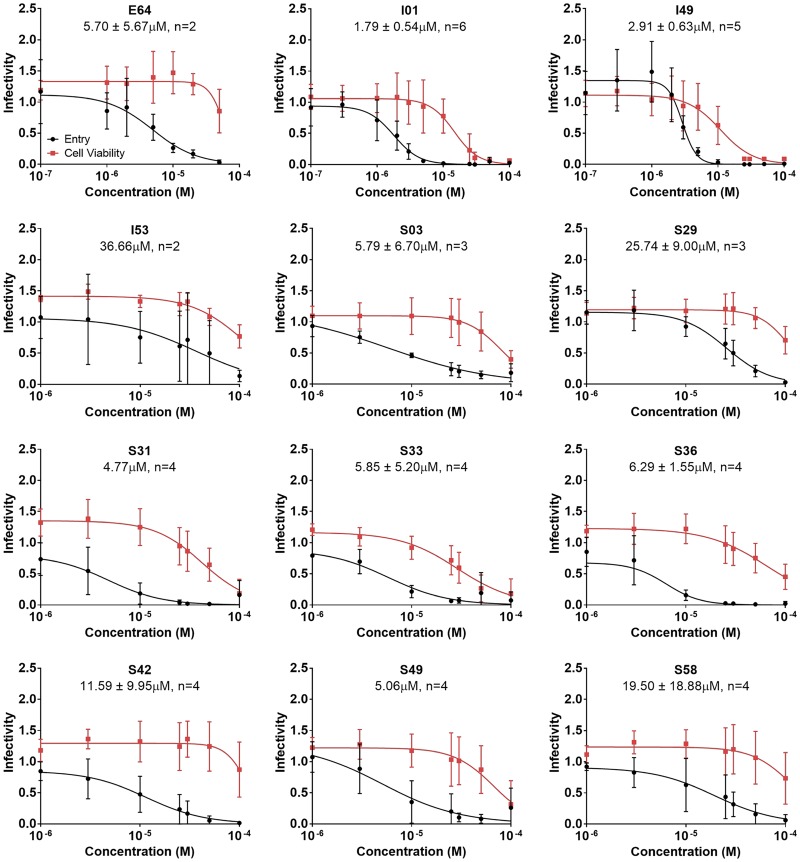
Dose-dependent activity and cytotoxicity for the most promising candidates against EBOV-pseudotyped virus. Activity (black) and cytotoxicity (red) are shown for the most promising 11 out of 25 compounds tested (*n* ≥ 2) from Fig. 3. Molecules from the initial and secondary screens are labeled with the prefixes I and S, respectively. Computed IC_50_ values are also shown along with the number of biological replicates used to calculate the viral entry results, with the standard error representing the 95% confidence interval for the IC_50_.

**FIG 6:**
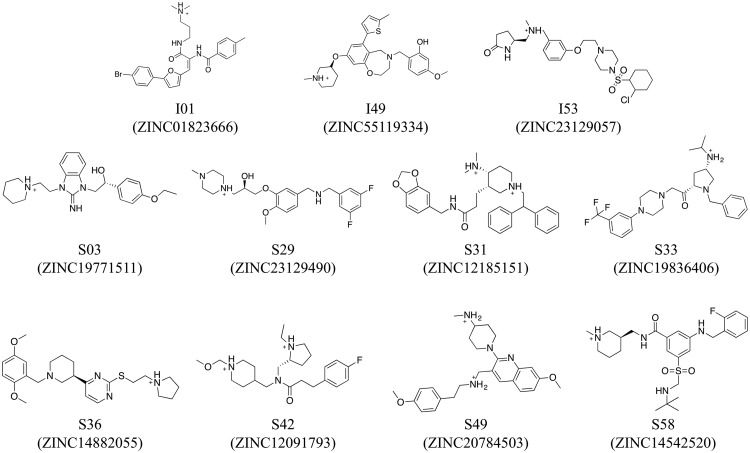
Structures of the 11 most promising candidates. Compounds from the initial screen (prefix I) and similarity screen (prefix S) are depicted with their corresponding name and ZINC identifiers.

To further explore the 25 most promising candidates identified from the two *in silico* screens (9 initial plus 16 secondary), in terms of reducing infectivity and their effects on cell viability, the dose-dependent activity for each was measured. Of the 25 tested from Fig. 3, 11 compounds exhibited generally well-behaved entry inhibition compared to that of the known control inhibitor, E64, seemingly independent of cytotoxicity, especially at the observed 50% inhibitory concentration (IC_50_) values, as shown in Fig. 5. The structures of the 11 compounds, with code names, are shown in Fig. 6.

Encouragingly, of the 11 molecules, 7 exhibited IC_50_ values under 10 μM, comparable to the results observed for the control inhibitor E64 (IC_50_ = 5.70 ± 5.67 μM) under the same conditions (Fig. 5 and Table 2). Specifically, the IC_50_ values for I01, I49, and S31 were less than 5 μM, and the IC_50_ values for S03, S33, S36, and S49 were less than 10 μM (Fig. 5 and Table 2). An accurate cytotoxic concentration that results in 50% cell death (CC_50_) could be obtained for 9 of the 11 compounds. For S42, S58, and E64, the computed CC_50_ values had large standard deviations, although examination of the cytotoxicity curves suggests minimal impact on cell viability. The two most potent molecules in this assay, I01 and I49, displayed CC_50_ values of approximately 11 to 15 μM (Table 2). All other hits had observed CC_50_ values of 29 μM or greater. Selectivity index (SI = CC_50_/IC_50_) values were also calculated. The higher the SI ratio, the more potent and the safer the compound is projected to be *in vivo*. Examination of the data showed a range of SI values from 3 to 14 for pseudotyped virus (Table 2). Of the compounds with computable SI, the two hits with the greatest SI were S03 and S49, which have SI values around 13 (Table 2).

**TABLE 2:** IC_50_, CC_50_, and SI for inhibitors of both *in vitro* EBOV particles

Molecule^*a*^	Pseudotyped			trVLP		
	IC_50_ (μM)	CC_50_ (μM)	SI^*b*^	IC_50_ (μM)	CC_50_ (μM)	SI^*b*^
E64	5.70 ± 5.67	58.31 ± 12364		5.77 ± 3.20	42.18 ± 18.25	7.3
I01	1.79 ± 0.54	14.64 ± 5.86	8.2	1.10 ± 0.99	9.72 ± 3.23	8.8
I49	2.91 ± 0.63	10.95 ± 6.46	3.8	2.67 ± 1.49	17.28 ± 2339	
I53	36.66	110.20 ± 57.97	3.0	13.64 ± 12.15	47.49 ± 31.84	3.5
S03	5.79 ± 6.70	80.03 ± 45.48	13.8	4.04	57.65 ± 24.16	14.3
S29	25.74 ± 9.00	113.10 ± 82.25	4.4	26.34 ± 17.13	75.10 ± 24.35	2.9
S31	4.77	42.37 ± 15.40	8.9	2.41	26.67	11.1
S33	5.85 ± 5.20	29.02 ± 12.39	5.0	3.06	17.50	5.7
S36	6.29 ± 1.55	66.93 ± 33.30	10.6	3.19	16.49	5.2
S42	11.59 ± 9.95	120.10 ± 4325		11.05 ± 12.93	123.50 ± 71.2	11.2
S49	5.06	66.60 ± 33.95	13.2	3.81 ± 3.16	22.38	5.9
S58	19.50 ± 18.88	118.60 ± 13353		4.08 ± 0.88	105.20 ± 57.14	25.8

a Prefix I, initial screen; prefix S, similarity screen.

b SI = CC_50_/IC_50_.

### Candidate compounds show improved or comparable inhibition of EBOV trVLPs.

**FIG 7:**
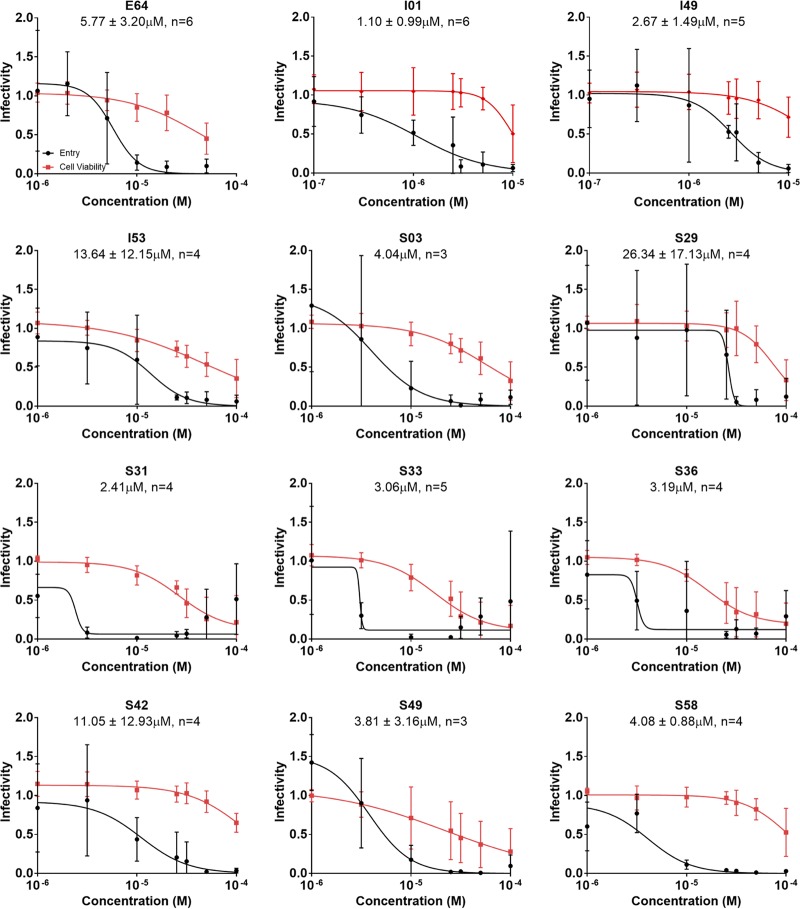
Dose-response infectivity of EBOV trVLPs with the treatment of 11 hits. The 11 most promising candidates identified from assays using pseudotyped virus were retested against EBOV trVLPs. Molecules from the initial and secondary screens are labeled with the prefixes I and S, respectively. Dose-response curves (black) and cytotoxicity results (red) were generated from replicate experiments (*n* ≥ 3). IC_50_s are displayed above each graph with the number of biological replicates performed to calculate the viral entry results.

To test the effects of the inhibitors in an EBOV system that utilizes virus particles of a size and shape similar to that of native EBOV (57), the 11 compounds were assessed for inhibitory effect against the EBOV trVLP system at various concentrations (Fig. 7). Notably, 8 of the 11 yielded IC_50_s under 5 μM (Table 2). Of particular interest, comparative linear regression analysis between the IC_50_s observed for each candidate against EBOV-pseudotyped virus and trVLPs yielded an *r*^2^ value of 0.55 (*n* = 12), which increased to 0.98 (*n* = 10) with the removal of the outliers I53 and S58 (Table 2). As expected, based on the good correspondence between the two dose-response assays, I01 remained the most potent compound, with an IC_50_ of 1.10 ± 0.99 μM (Fig. 7 and Table 2). Furthermore, S29, which exhibited an IC_50_ of approximately 26 μM in the pseudotyped experiment, was the only compound found to have an IC_50_ greater than 20 μM. The range of SI values from the trVLP experiments was between 2.9 and 25.8, with five candidates yielding selectivity indices greater than that of E64 (Table 2). Of the aforementioned five inhibitors, the two compounds with the largest SI values are S03 (14.3) and S58 (25.8) (Table 2). In summary, the results indicate good reproducibility between pseudotyped virus and trVLP assays, affirming the observed activity of the tested hits.

### Specificity of candidates for EBOV-GP.

The 11 hits were also examined using computational and experimental methods to ascertain if the observed activity involved nonspecific effects as a result of colloidal aggregation, pan-assay interference compound (PAINS) liabilities (58–60), or promiscuity. As an initial step to assess whether activity was a result of colloids, the compounds were screened for structural similarity to known aggregators using Aggregation Advisor (http://advisor.bkslab.org) (61). Eight candidates exhibited no known similarity to compounds in the current database. The three remaining compounds (I01, S29, and S31) were found to have 75%, 70%, and 77% structural similarity to a known aggregator. As described by Irwin and Shoichet (60), the addition of detergent should lead to a decrease in activity if a compound inhibits exclusively due to colloidal aggregation. Thus, activity was also tested in the presence of 0.025% Tween 80 (Table 3). Compounds here were defined as not sensitive to detergent if their IC_50_ values with and without detergent were similar, if their IC_50_ ranges with and without detergent overlapped, or if their activity increased. Based on these criteria, none of the hits appeared to be sensitive, although S49 was classified as ambiguous due to the absence of a computable error associated with the IC_50_ value.

**TABLE 3:** Summary of colloidal aggregation, detergent sensitivity, PAINS, and promiscuity alerts

Molecule^*a*^	Aggregation alert status (%)	IC_50_ (μM) with:		Detergent sensitivity	Alert	
		No detergent	Tween 80		PAINS	Promiscuity
I01	75	1.79 ± 0.54	2.48 ± 1.35	No	No	Yes
I49	No	2.91 ± 0.63	3.12 ± 0.68	No	Yes	No
I53	No	36.66	35.26	No	No	No
S03	No	5.79 ± 6.70	5.21	No	No	No
S29	70	25.74 ± 9.00	5.03 ± 3.62	No	No	No
S31	77	4.77	4.68 ± 0.36	No	No	No
S33	No	5.85 ± 5.20	8.12 ± 2.37	No	No	No
S36	No	6.29 ± 1.55	4.83 ± 1.11	No	No	No
S42	No	11.59 ± 9.95	36.52 ± 16.58	No	No	No
S49	No	5.06	17.77 ± 10.61	Ambiguous	No	No
S58	No	19.50 ± 18.88	40.51 ± 24.45	No	No	No

a Prefix I, initial screen; prefix S, similarity screen.

The 11 compounds were also subjected to an evaluation for PAINS alerts using 3 distinct computational filters (CBLigand [62], FAFdrugs3 [63], and SwissADME [64]). I49 was the only compound with a PAINS warning, which occurred for all three programs due to the possibility of Mannich reaction (64). Despite this warning, we opted to retain compound I49 at this early stage given the fact that multiple FDA-approved drugs elicit PAINS alerts (60). Finally, PubChem (65) was searched to assess if any of the compounds were previously reported as being active against multiple targets (i.e., whether or not they were promiscuous inhibitors). Results were only available for I01, which had been tested in 708 independent studies. In these prior works, I01 was reported as active in 14 studies to different targets, as an inconclusive inhibitor in 11 experiments, and as a nonspecific inhibitor of steroidogenic acute regulatory protein (BioAssay AID 651611; https://pubchem.ncbi.nlm.nih.gov/bioassay/651611) (66). Due to its apparent promiscuity, I01 was not considered further.

**FIG 8:**
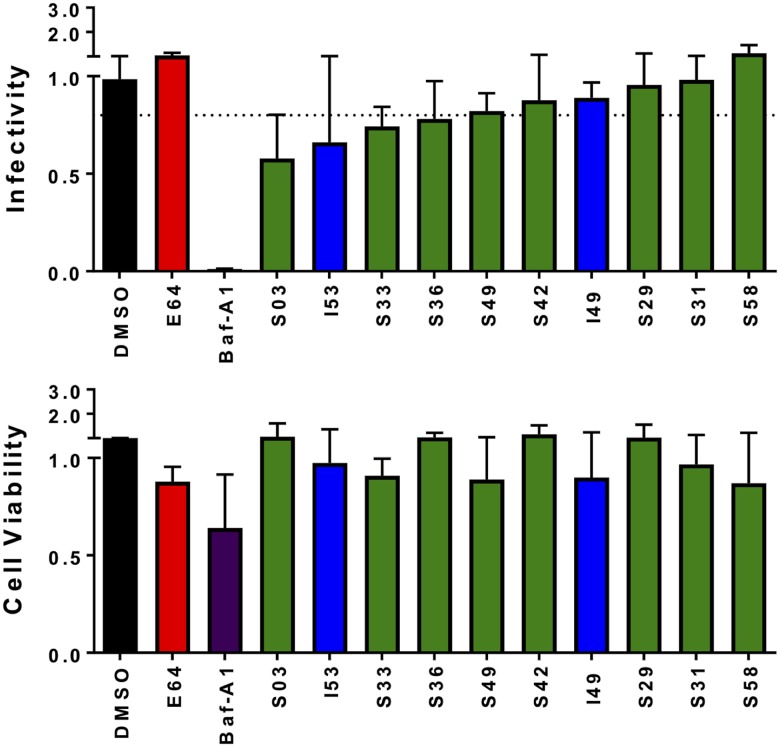
Infectivity of candidate compounds on VSV-G-pseudotyped virus entry ordered by infectivity. Compounds were tested against VSV-G-pseudotyped virus entry in triplicate (*n* = 3), at 10 μM (I49) or 25 μM (all others), based on cytotoxicity determined in the initial EBOV-pseudotyped virus experiments (Table 1). Cells were also treated with DMSO (black) and E64 at 25 μM (red) as negative controls of inhibition and bafilomycin A1 at 0.25 μM (purple) as a positive control for inhibition. Data from initial (I) and similarity (S) screen molecules are shown in blue and green, respectively. The hashed vertical line in the infectivity graph represents normalized infectivity of 0.8 per cell.

A counterscreen using VSV (HIV-1/VSV-G) was performed to experimentally determine the specificity of the prioritized set of 10 compounds. In a procedure similar to that of the EBOV-pseudotyped virus screen (Fig. 3), cells were treated with DMSO, the EBOV inhibitor E64, the nonspecific endosome acidification inhibitor bafilomycin A1 (67), or the candidates (Fig. 8). Compound I49 was tested at 10 μM due to its low CC_50_ (Table 2), while the other 9 candidates were tested at 25 μM. Notably, all compounds showed less inhibitory activity against the VSV-G screen (Fig. 8) than the initial EBOV-GP screen (luciferase signal, ≤0.25) (Fig. 3). The four compounds with the least average inhibitory activity against VSV-G and, therefore, likely higher specificity for EBOV were I49, S29, S31, and S58 (Fig. 8). These hits showed minimal effects on cell viability. Based on the aforementioned analysis, although other compounds shown in Fig. 8 would also be promising to explore, at this stage only I49, S29, S31, and S58 were selected for further characterization.

### Candidate compounds exhibited maximal inhibition postattachment and before membrane fusion.

**FIG 9:**
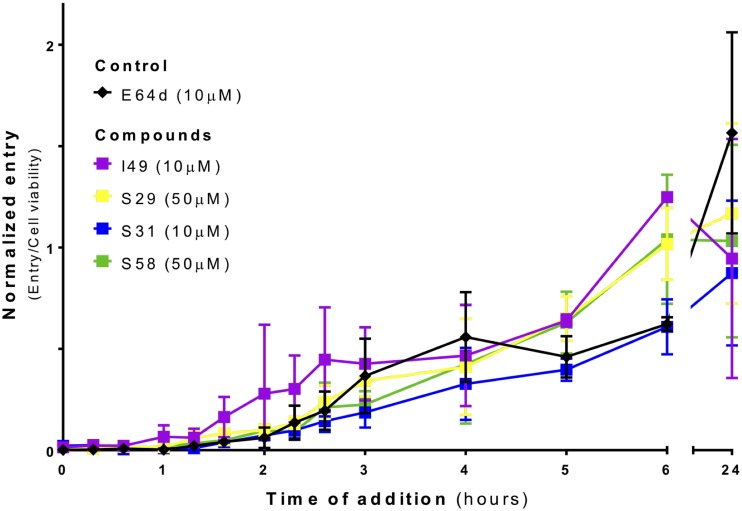
Candidate compounds act with the timing of E64d in EBOV entry. Time-of-addition experiments were performed with EBOV-pseudotyped virus on 293T cells. Candidate compounds (I49, S29, S31, and S58) and the control (E64d) were added at the indicated times of infection. The data shown are from two biological replicates that were performed in triplicate and normalized as described in Materials and Methods.

To explore the stage in the EBOV entry cascade at which the candidates act, time-of-addition (TOA) experiments (6, 30, 31, 68) were performed (Fig. 9) for the four compounds showing the most specificity, as suggested by the averaged activity results depicted in Fig. 8. In this TOA assay, 293T cells were treated with the four candidates and the cathepsin inhibitor E64d at various time points postinfection. Compounds were tested at the concentration required to reach maximum inhibition without a significant effect on cell viability as described by the dose-response curves against pseudotyped virus (Fig. 5 and Table 2). Importantly, the four candidate molecules exhibited an activity trend similar to that of the known control E64d, where maximum inhibition occurred up until the 80-min time point and then began to decrease (Fig. 9). The fact that the compounds track with E64d suggests they act after pinocytosis, after cleavage to the NPC1 binding form, but prior to the fusion step, as expected for molecules targeted to disrupt the interaction between the CHR and NHR necessary for 6HB formation.

### I49 and S31 exhibit reproducible pose stability in MD simulations.

Experimental characterization through concentration-dependent analysis, counterscreening, and TOA experiments suggested that I49, S29, S31, and S58 were the most potent, specific inhibitors of the premembrane fusion stage of EBOV entry identified from virtual screening. To more fully explore the energetic and geometric compatibility of these inhibitors with GP2 at the proposed pocket, all atom MD simulations of the DOCK-predicted poses were executed. As previously described (45, 55, 69), six replica 20-ns simulations for each candidate-GP2 complex were performed in explicit solvent, where each replica employed a different random seed. Ligand movement was quantified using RMSDs (root mean squared deviations) that accounted for translation, rotation, and differences in internal geometry relative to the initial predicted pose.

**FIG 10:**
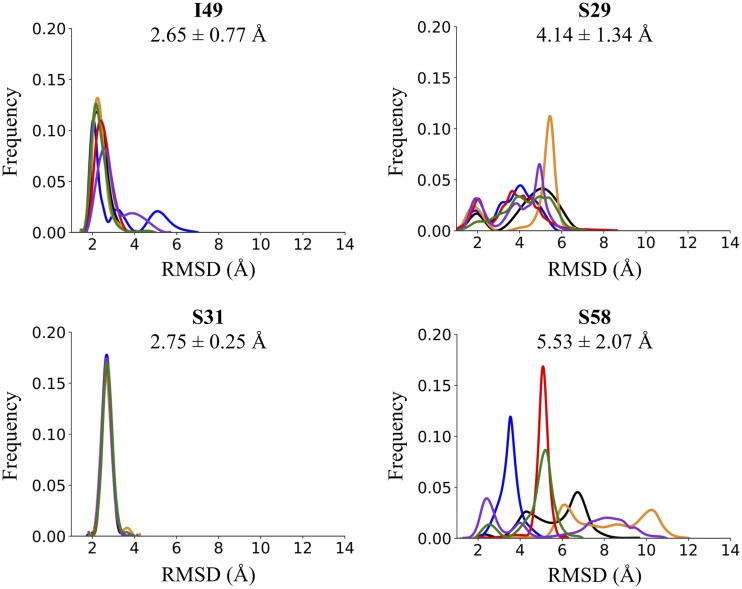
Variation from DOCK-predicted poses. Histograms of ligand RMSD computed from six replica MD simulations for the 4 hits. RMSDs (Å) include ligand pose variation in translation, rotation, and internal geometry relative to the initial DOCK pose. Average RMSDs are displayed below each molecule name with the position indicated by a gray dotted line.

Analysis of the trajectories showed that of the four compounds simulated, I49 and S31 maintained their DOCK-predicted poses more closely across all six simulations, as observed by the reproducible average RMSDs of 2.65 ± 0.77 Å and 2.75 ± 0.25 Å, respectively (Fig. 10). Since the average RMSDs of I49 and S31 were less than or equal to 2.75 Å, which is close to the typical benchmark (2.0 Å) commonly used in redocking validation tests (42), additional characterization for these two compounds was performed as described further below. In contrast, S29 and S58 adopted a wider variety of ligand poses during MD simulations, resulting in a larger range of RMSDs (Fig. 10). Visual inspection showed S29 adopted two overall geometries during its MD simulations, one closer to the original DOCK pose, which contributed to its bimodal RMSD histogram (Fig. 10). In general, compound S58 showed a much larger overall spread in RMSDs (mean of >5.5 Å) as a result of larger changes in internal geometry and/or movement in the pocket.

### Footprint interaction analysis.

**FIG 11:**
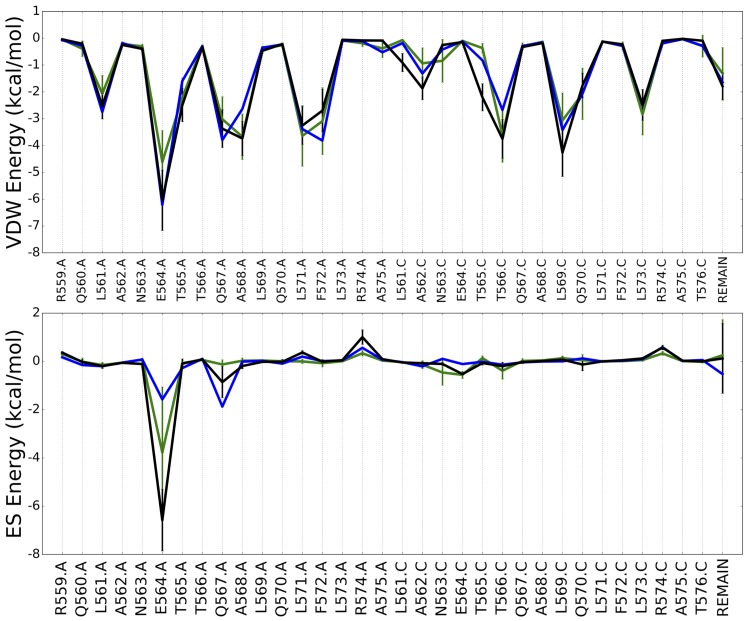
MD average footprint profile. VDW (top) and ES (bottom) energy footprint score for the reference (in blue), I49 (in green), and S31 (in black). Both figures show the top 33 residues with a high interaction energy and the remainder of the protein reference, named Remain. The energy for the reference ligand is derived from a minimized X-ray pose. The error bars represent the standard deviations from the means.

As a step toward understanding the hypothesized mechanism of action inhibiting six-helix bundle formation, the interactions of I49 and S31 with GP2 were characterized. To determine which residues had the greatest contribution to the ligand-receptor interactions across both hits, footprint interaction profiles were generated for each compound from the energies obtained over the MD trajectories (Fig. 11). Overall, the footprints showed striking similarity to the reference, especially in terms of the VDW profile (Fig. 11), suggesting good molecular mimicry of the CHR region. Moreover, I49 and S31 maintained strong contacts to a similar degree with the same residues, consistent with their overlap in the binding site and structural similarity. The residues with the most favorable interactions across the two candidates, which resulted in combined average energies greater than −2.5 kcal/mol, regarding VDW packing include (i) E564.A (−5.33 ± 2.35 kcal/mol), (ii) A568.A (−3.70 ± 0.75 kcal/mol), (iii) L571.A (−3.45 ± 0.96 kcal/mol), (iv) F572.A (−2.90 ± 1.06 kcal/mol), (v) T566.C (−3.69 ± 0.88 kcal/mol), (vi) L569.C (−3.66 ± 1.13 kcal/mol), and (vii) L573.C (−2.66 ± 0.70 kcal/mol) (Fig. 11). Regarding the ES energies, the reference profile contains two ES peaks corresponding to E564.A and Q567.A; however, E564.A was the only consensus residue with a combined average energy (−5.18 ± 2.55 kcal/mol) of less than −2.5 kcal/mol (Fig. 11). Notably, S31 also had a considerable interaction with Q567.A (−0.85 ± 0.64 kcal/mol) (Fig. 11). Further inspection of the individual footprint profiles of I49 and S31 showed that S31 interacted slightly more favorably with the EBOV five-helix bundle than I49 across multiple residues in addition to Q567.A. For instance, S31 had stronger predicted interactions with E564.A in both the VDW (−6.05 ± 1.12 kcal/mol) and ES (−6.58 ± 1.28 kcal/mol) plots than I49 (VDW, −4.62 ± 1.17 kcal/mol; ES, −3.78 ± 2.72 kcal/mol). Although simulation of S31 resulted in slightly greater energies over 6 of the 8 key residues (Fig. 11), the energies of the candidates are within one standard deviation from the means and therefore are insignificantly different, highlighting E564.A, A568.A, L571.A, F572.A, T566.C, L569.C, and L573.C as the key GP2 residues that interact with the reference ligand, I49, and S31.

**FIG 12:**
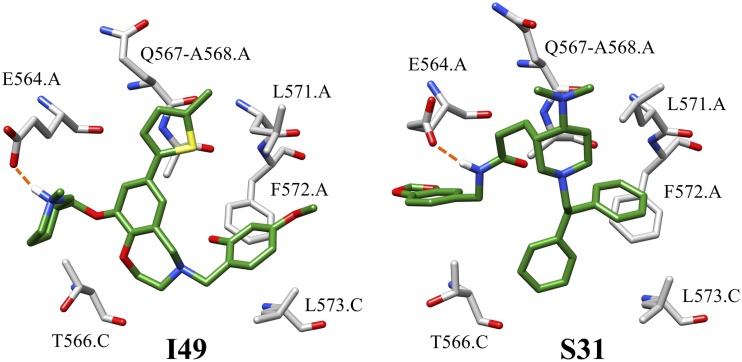
Compounds I49 (left) and S31 (right) in their predicted binding pose and location relative to key receptor residues. Hydrogen bonds are represented by an orange dashed line.

Of the corresponding residues, notable favorable VDW interactions were visualized at F572.A and T566.C. Specifically, F572.A was involved in strong nonspecific VDW interactions with the 4-methyloxy,6-carboxylphenyl substituent of I49 and the phenyl substituent of S31 (Fig. 12). Additionally, although both hits interact with T566.C, I49 was the only compound to exhibit a VDW interaction with T566.C throughout approximately 30.03% of the 6 simulations. Regarding ES interactions, the two inhibitors established and maintained strong ES contacts with E564.A across one main substituent throughout the majority of their MD simulations. For instance, the protonated nitrogen of the methylpiperidine substituent of I49 maintained water-mediated hydrogen-bonding interactions (∼25%) with the backbone and sidechain of E564.A and direct hydrogen-bonding interactions with the sidechain of E564.A about 32% of the time (Fig. 12). On the other hand, S31 retained water-mediated interaction with E564.A through approximately 28% of the simulations and direct hydrogen-bonding interactions for a total of approximately 57% of the simulations (Fig. 12). In summary, results suggest that I49 and S31 have the potential to establish and retain strong VDW and ES interactions with the predicted GP2 binding site.

### Sequence conservation across the key residues.

**FIG 13:**
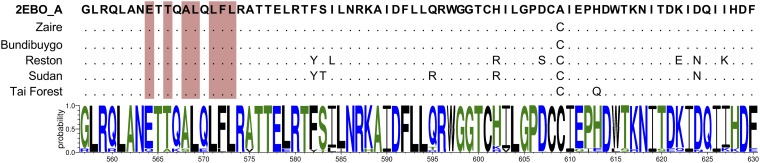
Sequence alignment of the core sequence of GP2. From top to bottom the representative sequences are, by GenBank accession number, 2EBO_A (Zaire), AHC70246 (Zaire, Mayinga 1976), YP_003815435.1 (Bundibugyo), APA16571.1 (Reston), ALL26375.1 (Sudan), and ALT19763.1 (Tai Forest). Below is the consensus sequence of the database of 581 fragmented or complete GP sequences generated by Weblogo3 (University of California, Berkeley). The 2EBO_A sequence begins at residue 557 and ends at residue 630. Alignment was performed with a Gap penalty opening of −25 and end-gap penalty of −15. The seven key residues in the protein sequence used for virtual screening and equivalent membrane fusion glycoproteins are highlighted in red. The dots signify sequence conservation.

To assess whether the inhibitors have the potential to interact favorably with other *Ebolavirus* species and related *Filoviridae* viruses, a comprehensive sequence alignment study was conducted. Specifically, 811 human sample sequences containing the complete GP genome for the five known *Ebolavirus* species, Zaire, Bundibugyo, Reston, Sudan, and Tai Forest, were selected via the Virus Pathogen Resource (ViPR) database (www.viprbrc.org; NIH). An additional 285 virus sequences were selected using BLAST (70) based on similarity to the core GP2 sequence (PDB entry 2EBO_A) used to conduct the virtual screens. Multiple-sequence alignment was then performed using COBALT (71) to align the above-mentioned 1,096 GP2-containing sequences to the full-genome sequence of GP2 (Zaire ebolavirus strain Mayinga-76; GenBank accession number AHC70246). Ultimately, 581 sequences seen in humans and nonhuman primates were retained with fragmented or complete GP sequences, which were used for sequence comparison analysis (Fig. 13).

Consistent with previous studies (29, 32, 72), there is high sequence identity for the core region of GP2 (G557-F630), with the exception of an intentional Cys609Ala mutation introduced into 2EBO_A to facilitate crystallization (25). Comparison of 581 genomes to a complete GP2 sequence (residues 502 to 676) exhibited approximately 90% conservation (Fig. 13, bottom). Notably, the 487 Zaire sequences, which are the most common and pathogenic (5–7), showed 97% sequence similarity. The seven key residues identified from the MD-based footprint analysis of I49 and S31 with GP2 (E564.A, T566.C, A568.A, L569.C, L571.A, F572.A, and L573.C) showed greater than 90% conservation across all 581 sequences, and for Zaire in particular there was ∼99% conservation.

Overall, the high sequence conservation among the subset of surveyed genomes for the five *Ebolavirus* species, for which a representative example is shown in Fig. 13, suggests that I49 and S31 have the potential to interact with the seven key residues in analogous GP2 binding sites (Fig. 13, shaded bars) and thereby inhibit sequence variants of Zaire ebolavirus and different *Ebolavirus* species. However, experimental testing would be required to characterize the activity of the small molecules against the different viruses.

## DISCUSSION

EBOV particles enter the cell through macropinocytosis (16), where they are later trafficked to the endosome and a conformation change is induced in the viral envelope protein GP2 that leads to membrane fusion (17–20). During this conformational change, the three CHR regions bind to the NHR trimer, forming a six-helix bundle (6HB) and mediating host-virus membrane fusion (25). Due to the current lack of FDA-approved therapeutics available to treat EVD and the key involvement of GP2 in virus entry, this study focused on the identification of small-molecule leads to inhibit the formation of the 6HB necessary for virus entry by targeting GP2 at the interface where the CHR interacts with the NHR. It is important, however, to note that our GP2 docking model is only an approximation of the EBOV prehairpin and, thus, is not likely to reflect all of the subtleties inherent in the actual biological system. Nevertheless, as the approach was successfully used by our group in prior work (45, 46, 73) and led to the identification of entry inhibitors targeting HIV gp41, we believe that adapting the methods to target Ebola is a reasonable strategy.

In this work, an initial virtual screen followed by a second similarity screen were performed to prioritize molecules with energetically favorable interactions with the GP2 NHR pocket. This led to a total of 165 compounds for experimental testing, of which 25 appeared promising in an EBOV-pseudotyped virus entry assay. Subsequent dose-response analyses narrowed down the group to 11 inhibitors with low to moderate cytotoxicity. To further validate activity, the hits were tested against EBOV trVLPs, which are more similar in shape and size to the native virus. The trVLP results correspond well with those obtained using pseudotyped virus, affirming the hits are promising EBOV inhibitors. To probe specificity, the hits were also tested using VSV-G-pseudotyped virus-like particles (Fig. 8). At this stage, four compounds (I49, S29, S31, and S58) were prioritized for additional analysis given their strong inhibition, low cytotoxicity, and apparent specificity for EBOV.

In the time-of-addition assay, the control curve for E64d showed the maximal level of inhibitory activity occurring between time 0 and up to the 80-min time point at which inhibition starts to decrease (Fig. 9). This is consistent with other studies (68) that have a lag in the EBOV entry pathway compared to that of influenza virus due to trafficking to the late endosome/lysosome. The timing of loss of inhibition of E64d and the experimental compounds, as reported in Mingo et al. (68), occurred with full restoration of infectivity by the 3-h time point. In contrast, full infectivity was not restored in our system until approximately 6 h. This could be due to differences in VLPs, cell types, or the readout assay. Importantly, all four hits (I49, S29, S31, and S58) exhibited a time-of-addition trend similar to that of E64d, suggesting that they are acting late in entry at a step that is after NPC1 binding.

Although the hits are hypothesized to prevent the collapse of the metastable intermediate into the stable 6HB, it is possible they interact with an earlier GP2/GP1 prefusion conformation. They could also disrupt interactions with other partner proteins, the lipid bilayer, or bilayer components or disrupt the putative E64d-sensitive cleavage step (68). Additional mechanistic investigation, such as site-directed mutagenesis and structural studies, will be required to confirm our hypothesis that the hits are inhibiting 6HB formation. Further study of the candidate compounds and future work on analogs and additional target sites could uncover important details about the fusion trigger. As an initial step, to help validate that the inhibitors prevent 6HB formation, the steric and energetic compatibility of the hits were explored via MD simulations. For the two hits with the most reproducible ligand poses (lower RMSDs), the MD analysis identified seven key GP2 residues (E564.A, A568.A, L571.A, F572.A, T566.C, L569.C, and L573.C) engaged in significant favorable protein-ligand interactions (Fig. 11). Notably, these residues are highly conserved (Fig. 13) across different *Ebolavirus* species, suggesting the hits have the ability to inhibit different types of EVD-causing viruses.

The compounds identified in this work have efficacy similar to that of other reported inhibitors of virus entry. Specifically, we identified 7 compounds with IC_50_ values of less than 10 μM, and three of the hits had IC_50_ values of less than 5 μM. Previously reported inhibitors include ZMapp, which is a combination of three antibodies, two of which appear to prevent conformational changes in the NPC-1-primed GP that are necessary for progression to late-stage entry (74). The estimated IC_50_ value for ZMapp is 5 to 10 μM (estimated from literature values reported by Holtsberg et al. [75] of 0.75 to 1.5 μg/ml). Other examples include C-peptide inhibitors (76) designed on the concept of the successful HIV peptides T20 (enfuvirtide) and C34, which prevent 6HB collapse (77). In contrast to HIV C-peptides, EBOV C-peptides showed weak or insignificant antiviral activity due to their inability to access the endosomal compartment (76). However, inhibition was significantly improved when researchers added the HIV Tat protein transduction domain (PTD), for which the resulting Ebo-Tat hybrid showed 99% inhibition at 75 μM (76). Other peptide-based inhibitors include prehairpin intermediate mimics reported by Clinton et al. (28), which showed mid-nanomolar inhibition in a pseudotype assay and a series of cyclopeptides (78) with IC_50_ values ranging from 3.2 to 5.9 μM.

In terms of small molecules, Basu et al. (6) reported a benzodiazepine derivative hypothesized to bind in a pocket observed in a prefusion conformation of GP1/GP2 that inhibited entry with an IC_50_ of 12.1 μM. Another study identified that the G protein-coupled receptor (GPCR) antagonist benztropine inhibited EBOV with an IC_50_ of 3.7 μM. Subsequent crystallographic studies by Stuart and coworkers (32, 35) showed that benztropine and other compounds, including bepridil, paroxetine, sertraline, toremifene, and, interestingly, ibuprofen, bound to the GP1/GP2 site and are thought to destabilize the protein complex (35). In contrast, the present compounds are hypothesized to stabilize a GP2 fusion intermediate, which prevents conformational changes required for formation of the 6HB. Notably, an investigation of drug synergy reported by Dyall et al. (79) using FDA-approved drugs showed that the majority of pairs identified as synergistic inhibitors of Ebola virus included an entry inhibitor. This suggests it is worthwhile to determine if there is synergy between the entry inhibitors identified in this work and other compounds.

In summary, this study has demonstrated the utility of computer-aided modeling, in conjunction with experimental testing, to identify four compounds (I49, S29, S31, and S58) that appear to be specific inhibitors of EBOV entry. We targeted a previously unexploited site on EBOV GP2 in a conformation representative of a prehairpin intermediate and utilized protein mimicry to select for small-molecule GP2 mimics. The identified inhibitors, hypothesized to prevent formation of the critical 6HB, serve as proof of principle for this technique and as a starting point for further GP2-targeted studies.

## MATERIALS AND METHODS

### Computational methods.

In this work, several computational methods were employed to target GP2, which can be arranged into five distinct protocols: (i) GP2 binding site and reference ligand designation through hot-spot identification, (ii) receptor and reference preparation, (iii) DOCK receptor setup, (iv) DOCK virtual screening protocols and compound prioritization, and (v) MD simulations. The work employed several software packages, including *antechamber*, *tleap*, *cpptraj* (80), *sander*, and *pmemd* from the AMBER suite of programs (University of California San Francisco) and *dms*, *grid* (81), and *sphgen* (82), which are part of the DOCK suite of programs (University of California San Francisco).

### Summary of approach for identification of CHR molecular mimics.

As described below, virtual screening was conducted for a high-resolution postfusion crystal structure of GP2 with one C-terminal heptad repeat (CHR) removed (termed the five-helix bundle). To identify molecules to compete with the removed CHR, docked ligands were characterized for their ability to mimic the interaction energetic patterns (footprints) (83) made by key CHR residues from chain C (I619.C to I626.C) with the N-terminal heptad repeat (NHR) trimer (discussed below). The procedure compares the VDW and ES interactions of a reference ligand (derived from key CHR residues) with a docked ligand (obtained from virtual screening) with each residue of the five-helix bundle to generate a footprint similarity score based on the Euclidian distance between the two interaction patterns. This similarity score, in combination with energy scores, was used to prioritize docked molecules for purchase and experimental characterization. We hypothesize that molecules that have interactions similar to those of the reference ligand will have an increased probability of serving as effective molecular mimics.

### GP2 binding site and reference ligand designation.

**FIG 14:**
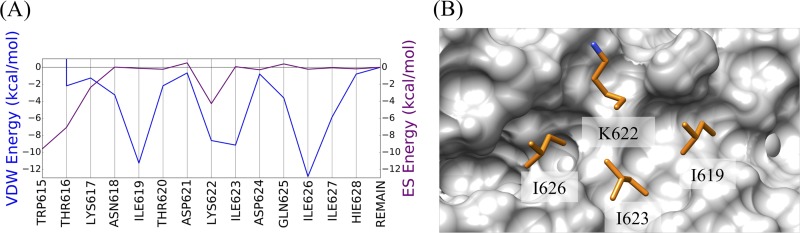
Reference ligand selection. (A) VDW (in blue) and ES (in magenta) footprint profiles for a subset of CHR peptide residues with the GP2 five-helix bundle. (B) Key reference ligand residues (orange) in the targeted protein binding site (gray).

By following previous protocols employed to target HIV gp41 (45, 73), hot-spot residues at the interface of the CHR (residues 599 to 632) and NHR (residues 556 to 599) of GP2 were identified in the postfusion X-ray structure (PDB entry 2EBO [25]) through examination of molecular footprints (84). Strong favorable van der Waals (VDW) and electrostatic (ES) interactions present in the 6HB were used to identify a promising binding site for virtual screening and a subset of CHR residues to aid in the selection of small-molecule mimics. The AMBER14 accessory program *tleap* was used to protonate the 2EBO X-ray structure and assign the ff99SB protein force field (85). A three-step minimization protocol was employed to relax the coordinates using the AMBER14 *sander* module, where 100 cycles were completed in turn with decreasing heavy-atom restraints of 1,000, 100, and 10 kcal mol^−1 ^Å^−2^, respectively. As shown in Fig. 14A, the CHR residues with the most favorable VDW interactions in the energy-minimized structure include I619.C, K622.C, I623.C, and I626.C, and the residues with the most favorable ES interactions are W615.C, W616.C, and K622.C. Of the aforementioned residues, only K622.C was selected due to its central location in the pocket and strong hydrogen-bonding interaction with Q567.A of the adjacent chain’s NHR at its carbonyl oxygen. For simplicity, a continuous CHR peptide sequence in the range of I619.C to I626.C (Fig. 14B) was subsequently chosen as the reference ligand to prioritize molecules for purchase and to further define a narrow binding site for screening.

### Receptor and reference ligand preparation.

To create a suitable GP2 model for docking, residues A609 to F630 from the CHR of chain C were removed from the preminimized, crystallographic structure, resulting in a five-helix bundle with the previously designated binding pocket exposed. The CHR residues (I619.C to I626.C) that made the most favorable interaction with the five-helix bundle in footprint analysis were prepared by following standard docking preparation protocols (86, 87). Briefly, the reference ligand was manually isolated (Chimera [88]), protonated (MOE; Chemical Computing Group), and assigned AM1-BCC (89, 90) charges (*antechamber* program). To avoid introduction of artificial terminus charges due to fragmentation, the N and C termini of the reference peptide were capped with acetaldehyde (ACE) and *N*-methylamide (NME) groups, respectively, using *tleap*. AMBER14 was then employed to minimize the noncovalent complex following *tleap* assignment of the ff99SB (85) force field for the receptor and GAFF (91) force field for the reference. Subsequently, a short restrained 3-stage energy minimization was performed to relax the complex coordinates prior to saving separate receptor and reference files (MOL2 format) to be used for the virtual screening.

### DOCK receptor setup.

The GP2 receptor (five-helix bundle) was prepared for docking by following protocols outlined in Allen et al. (45). Briefly, a molecular surface of the 2EBO five-helix bundle was computed using the DOCK accessory program *dms*, followed by the generation of docking spheres using the program *sphgen* (82). Subsequently, docking grids were generated using the program *grid* (81) with 6 to 9 Lennard-Jones exponents for the intermolecular van der Waals energies and a ε = 4r distance-dependent dielectric to scale the intermolecular Coulombic energies. The calculations employed a 0.3-Å grid spacing, which extended 8.0 Å in all directions around the sphere set (86).

### DOCK virtual screening protocols and compound prioritization.

Following previously published protocols (45, 55), a screening library of 1,707,345 commercially available drug-like molecules from the ZINC database (92) (Chembridge vendor) was sorted according to the number of rotatable bonds and divided into 42 chunks of at most 50,000 molecules. Compounds were flexibly (FLX) (42) docked to the GP2 five-helix bundle in parallel, using the MPI version of DOCK6.6 (University of California San Francisco). For each docked compound, the best scoring pose was retained, which was then energy minimized using the standard DOCK Cartesian energy (DCE) function to further fine-tune the interactions between the receptor and candidate ligands and permit footprint similarity scoring (42, 84), where the similarity in VDW and ES interaction profiles between the reference and screened molecules was quantified using Euclidean distance.

Key descriptors were computed with the program MOE for the 100,000 top-scoring molecules based on DCE score, including the number of Lipinski violations, number of chiral centers, and logP, to aid in compound prioritization. The MOE MACCS clustering method was concurrently employed, using a best-first approach, to group compounds into structurally related families with the best DCE scored compound per family to serve as a clusterhead. To promote diversity in compound selection, the top-scored clusterheads were rank ordered using five distinct scoring criteria: (i) the sum of the van der Waals and electrostatic DOCK Cartesian energy score (DCE_SUM_), (ii) the van der Waals FPS score (FPS_VDW_), (iii) the electrostatic FPS score (FPS_ES_), (iv) the sum of the FPS_VDW_ and FPS_ES_ scores (FPS_SUM_), and (v) the combined DCE_SUM_ and FPS_SUM_ scores (total score, or TS) (45). Following 3D visual inspection of the top-scoring members from each of the five lists, 83 compounds, referred to with the prefix I (initial screen), were purchased for experimental testing. A second set of 82 ligands, referred to with the prefix S (secondary screen), was purchased based on similarity comparisons to hits identified in the initial screen. Similarity was computed using the following DOCK6 scoring functions: Hungarian similarity (56), footprint similarity (83), pharmacophore similarity (93), and volume overlap. For both screens, additional ligand properties considered included central location in the pocket, number of chiral centers (less than 2), formal charge between −1 and +1, favorable overall score with respect to the particular rank-order method, and favorable electrostatic score.

### MD simulations and analysis.

For the most promising candidates, MD simulations were performed to assess geometric and energetic stability. The AMBER14 accessory programs *antechamber* and *tleap* were used to protonate, solvate, assemble, and assign force-field parameters for the protein receptor (ff14SB) (94), solvent (TIP3P) (95), and ligand (GAFF) (91). Ligand partial charges were obtained from those preassigned by the ZINC database (92). The five-helix bundle was capped where the N terminus was capped with ACE and the C terminus was capped with NME.

As previously described (69), a nine-step protocol was used to equilibrate each solvated ligand-protein complex. Briefly, all simulations were performed using the CUDA-accelerated version of *pmemd* (96–98) in AMBER16. In short, first the solvent and protein-ligand hydrogens were minimized with a restraint weight of 20.0 kcal mol^−1 ^Å^−2^ on all complex heavy atoms for 10,000 cycles. Second, the restraint was lifted and the entire complex was minimized for 5,000 cycles. Third, over 250 ps, the system was heated from 50 to 300K. Fourth, a short MD simulation of 500 ps, with an all-atoms restraint weight of 20.0 kcal mol^−1 ^Å^−2^, was performed to optimize the water box density to 1. Lastly, each complex underwent five equilibration steps, each 200 ps in length, with lessening restraint weights of all protein and ligand heavy atoms. For the protein, the restraint weights were (i) 10.0, (ii) 5.0, (iii) 0.1, (iv) 0.1, and (v) 0.1 kcal mol^−1 ^Å^−2^, and for the ligand they were (i) 10.0, (ii) 5.0, (iii) 0.1, (iv) 0.1, and (v) 0 kcal mol^−1 ^Å^−2^. The equilibrated complexes underwent six replica MD simulations for 20 ns, with a restraint weight of 0.1 kcal mol^−1 ^Å^−2^ on protein heavy atoms. The equilibration and production runs were performed at a constant temperature of 300.0K.

Visualization of MD trajectories was conducted using VMD (99) and Chimera (88). The AMBER14 accessory program *cpptraj* (80) and in-house protocols were utilized to extract VDW and ES energies (with distance-dependent dielectric) and compute molecular footprints, RMSDs (root mean squared deviations), and hydrogen-bonding interactions of each compound throughout its MD trajectories (4,000 frames for each simulation). As previously described (45, 55), predicted interaction energies from all six replica MD trajectories were used to calculate the mean VDW and ES energies between the small molecule and each residue of the five-helix bundle. Residues with energies of less than −2.5 kcal/mol for the reference ligand and experimentally verified GP2 entry inhibitors were used to select key GP2 residues involved in an interaction energy. To compute ligand RMSDs, a two-step protocol was executed (73). First, the protein-ligand complex in each frame of the trajectory was aligned using *cpptraj* so that the protein’s alpha carbons overlapped. Second, atomic-level small-molecule translation and rotation compared to that of the docked pose was quantified. For interpretation, RMSDs were binned based on frequency using *cpptraj* and plotted using Python (Python Software Foundation). The AMBER accessory program *cpptraj* was used to extract the direct and water-mediated hydrogen-bonding interactions from each trajectory and provide a frequency, location, and frame.

### Experimental methods.

The experimental methods to characterize the inhibitory activity of the small molecules identified from *in silico* screening are described below. Three different assays were employed: (i) pseudotyped HIV-1/EBOV-GP was utilized to assess viral entry, (ii) pseudotyped HIV-1/VSV-G was utilized to assess inhibitor specificity, and (iii) EBOV trVLP was utilized as a second confirmatory assay of viral entry.

### Cell lines and plasmids.

The following reagents were obtained through the AIDS Reagent Program, Division of AIDS, NIAID, NIH: TZM-bl cells (number 8129; from J. C. Kappes and X. Wu) (100) and replication-defective HIV vector pNL4-3.Luc.R-E- (number 3418; from N. Landau) (101). The following reagent was obtained through BEI Resources, NIAID, NIH: vector pcDNA3.1 containing Zaire ebolavirus glycoprotein NR-19814 (102). Plasmid pCMV-VSV-G was a gift from E. Freed (NCI-Frederick). The EBOV trVLP transfection plasmids pCAGGS-VP30, pCAGGS-NP, pCAGGS-VP35, pCAGGS-L, pCAGGS-T7, p4cis-vRNA-Rluc, and pCAGGS-Tim1 were a gift from H. Feldmann (NIH) (57).

293T cells (ATCC CRL-11268) and TZM-bl cells were cultured in Dulbecco's modified Eagle's medium (DMEM; Corning) supplemented with 10% heat-inactivated fetal bovine serum (FBS; Gemini Bio-Products) containing 100 μg/ml of streptomycin and 100 U/ml of penicillin (DMEM–PS–10% FBS) in a 37°C incubator with 5% CO_2_ atmosphere.

### Pseudotyped HIV-1/EBOV-GP and HIV-1/VSV-G virus preparation and titration.

Replication-incompetent pseudotyped virus containing the replication machinery of HIV-1 and the outer glycoproteins of either Ebola (HIV-1/EBOV-GP) or vesicular stomatitis (HIV-1/VSV-G) virus were prepared by a standard transfection method using polyethylenimine (PEI) MAX 4000 (Polysciences) (103, 104). Specifically, 24 h prior to transfection, 3 × 10^6^ cells of 293T cells were seeded per 100-mm dish. The cells were cotransfected with equal amounts (7.5 μg) of HIV-1 core plasmid (pNL4-3.Luc.R-E-) and envelope protein plasmid using 45 μg PEI transfection reagent per plate. Twenty-four h posttransfection the medium was replaced, and pseudotyped virus was harvested from the supernatant at 48 and 72 h posttransfection. The supernatant was clarified by low-speed centrifugation followed by filtration with a 0.45-μm-pore-size filter (Millipore). The filtered supernatant was centrifuged (27,000 rpm) at 4°C for 2 h, and the pellet was resuspended in Dulbecco’s phosphate-buffered saline (DPBS) and stored at −80°C until needed (105). Infectious titers of virus stocks were quantified by 5-bromo-4-chloro-3-indolyl-β-d-galactopyranoside staining in TZM-bl cells (106, 107).

### EBOV trVLP preparation.

A transient-transfection-based transcription- and replication-competent system that models the entire replication cycle at biosafety level 2 was utilized to confirm inhibition. This system is more physiologically relevant than pseudotyped virus due to the native size and shape of the EBOV particles. Preparations of EBOV trVLPs were prepared as previously described (57, 108). Briefly, 293T cells were seeded in 2 ml in a 6-well plate at ∼50% confluence. Twenty-four h postseeding, the cells were transfected with the following plasmids per well: 75 ng pCAGGS-VP30, 125 ng pCAGGS-NP, 250 ng pCAGGS-T7, 125 ng pCAGGAS-VP35, 1 μg pCAGGS-L, and 250 ng p4cis-vRNA-Rluc, using 5.5 μg PEI transfection reagent. Twenty-four h posttransfection, medium was replaced with 4 ml DMEM–PS–5% FBS. Seventy-two h posttransfection, the supernatant containing the trVLPs was pooled, clarified by low-speed centrifugation, and stored at 4°C.

### Screening of *in silico*-selected compounds in viral entry assays.

Viral entry was measured using a luciferase reporter. Testing of selected compounds and controls against all three types of virus particles, EBOV (HIV-1/EBOV-GP) pseudotyped, VSV (HIV-1/VSV-G) pseudotyped, and EBOV trVLP, was performed in a similar procedure. 293T cells were seeded at 2 × 10^4^ cells/well in 96-well tissue culture-treated white-bottom plates (Greiner) that were precoated with 25 μg/ml linear PEI (Sigma). For EBOV trVLP infection, helper ribonucleoprotein (RNP) components must be provided in *trans* through expression plasmid transfection 24 h postseeding (amounts of helper RNP plasmids per well were 4.16 ng pCAGGS-VP30, 6.94 ng pCAGGS-NP, 6.94 ng pCAGGS-VP35, 55.55ng pCAGGS-L, and 13.88 ng pCAGGS-Tim1, with 262.41 ng PEI transfection reagent). Twenty-four h postseeding (pseudotyped virus particles) or posttransfection (trVLPs), 293T cells were pretreated with selected compounds or controls for 1 h at 37°C. The medium then was removed and the cells were infected with virus particles that had also been pretreated for 1 h at 37°C. After 2 h the inoculum was removed, the cells were washed briefly with PBS, and fresh medium was added. Plates were incubated for 48 h, and viral entry was measured using the luciferase reporter. The experiment was also performed in the absence of virus to determine the toxicity of the selected compounds and controls.

Viral entry and cell viability were measured using ONE-Glo + Tox Luciferase reporter and cell viability assay (Promega) according to the manufacturer’s protocol using a Spectra Max M5 plate reader (Molecular Devices). Luciferase signal was normalized to the cell viability and then further normalized to the luciferase signal in the DMSO-treated samples (45). Compounds with infectivity signal per cell as a fraction of the maximum below 0.25 were considered active hits in the initial screening. Additionally, for the dose-response assays, 50% inhibitory concentration (IC_50_), 50% cytotoxicity concentration (CC_50_), and 95% confidence intervals (CI_95_) were computed, and IC_50_ was plotted using Prism 7.0c (GraphPad Software, La Jolla California USA). CC_50_s were reported if a standard deviation within 2-fold of the CC_50_ could be calculated.

As previously described (6), selected controls were dissolved in DMSO. Cathepsin inhibitor E64 (Millipore) is a cysteine protease inhibitor that prevents cleavage events that are necessary specifically for EBOV fusion with the endosomal membrane. It is used as a positive control for inhibition in HIV/EBOV-GP and EBOV trVLP assays and as a negative control in VSV-G assays, as it does not inhibit VSV-G fusion. E64d has the same action as E64 but is cell permeable. Bafilomycin A1 (Calbiochem) is a vacuolar ATPase inhibitor that prevents both EBOV and VSV entry by alkalinizing the endosome and is used as a positive control for inhibition in both assays.

Cells were infected with either EBOV- or VSV-G-pseudotyped virus at a multiplicity of infection (MOI) of 0.1 or with 50 μl of EBOV trVLPs. Where indicated, 0.025% Tween 80 (Sigma) was also added to the assay to test for colloidal aggregation.

### Time-of-addition assay.

293T cells were seeded at 2 × 10^4^ cells/well in PEI-precoated 96-well tissue culture-treated white-bottom plates. The next day, EBOV-pseudotyped virus was added to the cells at an MOI of 0.1. The plates were centrifuged for 1 h at 4°C at 1,000 × *g* to allow the virus to attach to the cells and to synchronize the infection. The plates were washed with PBS to remove unbound virus. The plates were then moved to 37°C to allow for viral entry (0 h). Small molecules I49 (10 μM), S31 (10 μM), S29 (50 μM), and S58 (50 μM) and the E64d control (10 μM; Millipore) were added to the plates at various time points as indicated. Cell viability and viral entry were measured and analyzed 48 h postinfection as described above.
